#  A Simple and Rapid Protocol for Producing Yeast Extract from *Saccharomyces cerevisiae *Suitable for Preparing Bacterial Culture Media

**Published:** 2016

**Authors:** Omid Zarei, Siavoush Dastmalchi, Maryam Hamzeh-Mivehroud

**Affiliations:** a *Biotechnology Research Center, Tabriz University of Medical Sciences, Tabriz, Iran. *; b *Department of Pharmaceutical Biotechnology, Faculty of Pharmacy, Tabriz University of Medical Sciences, Tabriz, Iran. *; c *Student Research Committee, Tabriz University of Medical Sciences, Tabriz, Iran. *; d *Department of Medicinal Chemistry, Faculty of Pharmacy, Tabriz University of Medical Sciences, Tabriz, Iran.*

**Keywords:** Baker’s yeast, Yeast extract, Bacterial growth, *Saccharomyces cerevisiae*

## Abstract

Yeasts, especially *Saccharomyces cerevisiae,* are one of the oldest organisms with broad spectrum of applications, owing to their unique genetics and physiology. Yeast extract, i.e. the product of yeast cells, is extensively used as nutritional resource in bacterial culture media.

The aim of this study was to develop a simple, rapid and cost benefit process to produce the yeast extract. In this procedure mechanical methods such as high temperature and pressure were utilized to produce the yeast extract. The growth of the bacteria feed with the produced yeast extract was monitored in order to assess the quality of the product. The results showed that the quality of the produced yeast extract was very promising concluded from the growth pattern of bacterial cells in media prepared from this product and was comparable with that of the three commercial yeast extracts in terms of bacterial growth properties. One of the main advantages of the current method was that no chemicals and enzymes were used, leading to the reduced production cost. The method is very simple and cost effective, and can be performed in a reasonable time making it suitable for being adopted by research laboratories. Furthermore, it can be scaled up to produce large quantities for industrial applications.

## Introduction

Yeasts are important eukaryotic microorganisms belong to the kingdom of fungi. From historical point of view, they have been used for wide variety of applications since thousands of years ago such as fermentation, baking and bioremediation (1-3) as well as broad range of research activities in biological sciences owing to their unique genetics and physiology (4-8). In addition, nutrient cell content of yeasts known as “yeast extract” are also utilized for many important purposes. Yeast extract is a processed yeast product, which comprises the soluble components of yeast cells and is extensively used in food industries as food flavoring, additives and vitamin supplementsas well as nutritional resource for bacterial culture media used in microbiology and biotechnology (9-10). Moreover, the importance of using the yeast extract in industrial fermentation to produce the microbial biomass or products has been proven by numerous studies (11-14). Different yeast species especially *Saccharomyces cerevisiae *(known as baker’s yeast) have been employed for the production of yeast extract (15-17). Yeast extract is produced using variety of methods such as those based on autolysis and hydrolysis methods. The aim of different methods is to disrupt yeast cell wall (for releasing the cell contents) and recover the soluble fraction. Depending on the purpose of production, the procedure for obtaining the yeast extract varies. For instance, if the aim of yeast extract production is to use it in food or cosmetic industries, several steps are required to obtain the food/cosmetic grade yeast extract. These steps include plenty of physical, chemical and enzymatic methods for disrupting the yeast cell wall, followed by several additional steps such as filtration, chromatography or other separation methods for removing insoluble materials in order to obtain odorless, tasteless and colorless product (18-19). Although the quality of yeast extract is improved by applying these additional procedures but most of these steps are very laborious, time consuming, expensive and non-essential where the aim of production is to use the yeast extract for microbiological research as one of the main components of bacterial cell culture media. 

Regardless of availability of numerous commercial yeast extracts for the preparation of microbial culture media, a simple method for preparing the high quality yeast extract will be of great benefit. Taking into account the importance and wide range of application of yeast extract in microbial fermentation media, our aim in the current study, was to develop a simple, rapid and cost benefit process to produce the yeast extract applicable in different laboratories in order to meet the experimental needs as well to be used in industry as essential component of microbial fermentation media in large quantities. 

## Materials and Methods

*Preparation of the yeast extract*

Aqueoussuspension of yeast cells was prepared by adding 500 g of baker’s yeast (*Saccharomyces cerevisiae*) to 2 L of deionized water (milli-Q water). The prepared suspension was autoclaved at 115 °C for 10 min followed by fast cooling on ice. Then, the cell debris was separated by centrifugation at 400 gat 4 °C for 10 min. The supernatant was re-centrifuged again at the same condition to make sure of removing the insoluble cellcontents. Therecovered supernatant containing the soluble component was re-autoclaved and cooled again as described above. The produced water soluble extract was stored at –20 °C until preparation of the yeast extract in powder form. Subsequently, the soluble fraction was subjected to mini spray dryer (B-290, Büchi Labortechnik AG, Flawil, Switzerland) for obtaining the powder form of the yeast extract.

*Bacterial cell growth assessment*

The growth of the bacteria feed with the produced yeast extract was monitored in order to assess the quality of the product. To this end, different bacterial culture media either in the form of solid or liquid were prepared. In the first approach we used only yeast extract as the sole source of nutrient in bacterial cell culture media. For this purpose, solid and liquid media were prepared using the following protocol: For solid medium a mixture of 8g of the produced yeast extract powder and 15 g agar (Merck, Darmstadt, Germany) was dissolved in 1L deionized water and autoclaved (121 °C, 15 lb) for 15 min followed by dispensing in 8 cm sterile plastic plates. As a negative control medium, a similar solid medium without yeast extract powder was also prepared. For liquid medium, 20 g of produced yeast extract powder was dissolved in final volume of 1L deionized water and was autoclaved as mentioned above. The cooled media was dispensed in sterile tubes. A similar negative control medium (i.e. the media without yeast extract) was also prepared. The bacteria used for culturing in the prepared media were* E. coli *(DH5α) and *Staphylococcus aureus *(PTCC 1112) as gram negative and gram positive bacteria, respectively. For solid media, the bacteria were streaked out on the plate and incubated overnight at 37 °C. In the case of liquid media, a fresh overnight culture of bacteria was diluted 1:100 in medium and incubated at 37 °C while shaking at 180 rpm. At different time intervals (i.e. 2, 4, 8, 16, 24 and 48 h) samples were taken under sterile condition for monitoring the growth of bacteria in the prepared media by reading optical densities (OD) of the samples at 600 nm using UV spectrophotometer (Ultrospec 2000, Amersham Pharmacia Biotech). Each experiment was performed in triplicate.

As a further evaluation approach, the bacterial growth was monitored in Luria Bertani broth media (a commonly used medium in microbiology) prepared using the yeast extract produced in this study and those obtained from commercial suppliers. The LB broth medium was prepared as follows: 5 g of yeast extract powder, 10 g tryptone (HiMedia, Cat. No: RM014) and 5 g NaCl (Scharlau, Cat. No: SO0225) were dissolved in 1 L of deionized waterwith subsequent autoclaving and dispensing in sterile tubes. In order to compare the bacterial cell growth profile in medium prepared by our produced yeast extract with similar commercial yeast extract products, three different yeast extracts from well-known companies, namely, Sigma (Cat. No.: 92144-F), Merck (Cat. No:1.03753) and Applichem (Cat. No: A3732) were used. A medium containing all ingredients except yeast extract was prepared and used as negative control medium. All the procedures related to the bacterial culturing and monitoring of bacterial cell growth were conducted as described above.

*Product composition analysis*

Quantitative compositional analysis of produced yeast extract was performed by a references laboratory (ASA laboratory, Tehran, Iran). Total amount of protein, fat, ash, sodium chloride, total volatile nitrogen (TVN), moisture and pH were the chemical properties that were analyzed.

*Statistical analysis*

All the statistical analyses for comparing bacterial growth in LB broth media prepared using different yeast extracts were carried out using t-test implemented in SPSS program (version 21.0). A p-value of <0.05 was considered statistically significant. 

## Results

*Production of yeast extract*

In the current work, for the preparation of the yeast extract, we used baker’s yeast (*Saccharomyces cerevisiae*)*.* The aqueous suspension of yeast was prepared followed by autoclaving and centrifugation in order to obtain soluble fraction. These steps (centrifugation and autoclaving) were repeated one more time for the complete removal of the cell debris. The supernatant was applied to spray dryer to obtain powdered yeast extract.

*Assessment of bacterial cell growth in solid and liquid media*

**Figure 1:**
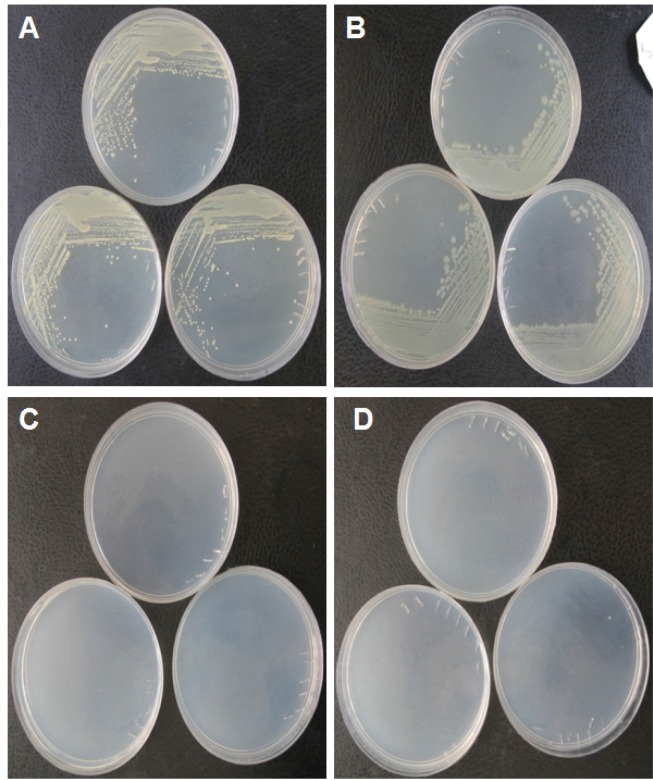
Growing bacteria on solid media prepared by the produced yeast extractas the sole source of energy for bacteria. (A) Colonies of *Staphylococcus* *aureus* as gram positive bacteria on the solid media prepared by the produced yeast extract. (B) Colonies of *E.* *coli* DH5α as gram negative bacteria on the solid media prepared by the produced yeast extract. (C and D) Yeast extract free solid media as control for culturing of *Staphylococcus* *aureus* and *E.* *coli* DH5α, respectively. As seen in the control plates, there are no colonies in the media. The experiments were conducted in triplicates.

**Figure 2:**
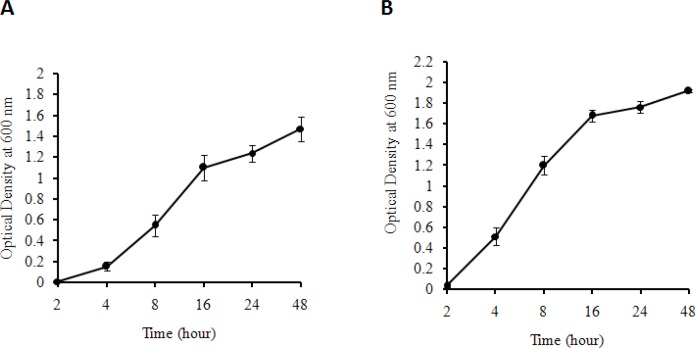
(A) Growth pattern of *E.* *coli* DH5αas gram negative bacteria (B) and *Staphylococcus* *aureus*as gram positive bacteria on liquid media prepared by the produced yeast extract (as sole source of energy) at different time intervals. As it is seen the number of bacteria increases during a 48 h period for both types of bacteria. The average ODs for three individual experiments at different times and the corresponding standard deviation are shown

*E. coli DH5α *and *Staphylococcus aureus*, as the representatives for gram negative and gram positive bacterial types, respectively, were utilized to systematically determine the bacterial growth in both solid and liquid media prepared by the produced yeast extract. The result showed that both bacteria can grow in the solid medium containing the produced yeast extract as the sole source of nutritional support. Figures 1a. and 1b. show the colonies of *E. coli DH5α* and *Staphylococcus aureus,* respectively. No colonies were observed for control media for both bacteria (Figures 1c. and 1d.). Figures 2a. and 2b. demonstrate the growth rate of *E. coli *DH5α and *Staphylococcus aureus *in liquid broth media composed of the produced yeast extract as a sole source of energy, respectively. The plots indicate that the quality of the yeast extract produced in this work is rich enough to be used as a sole source of nutrition for bacterial cell growth.

**Figure 3:**
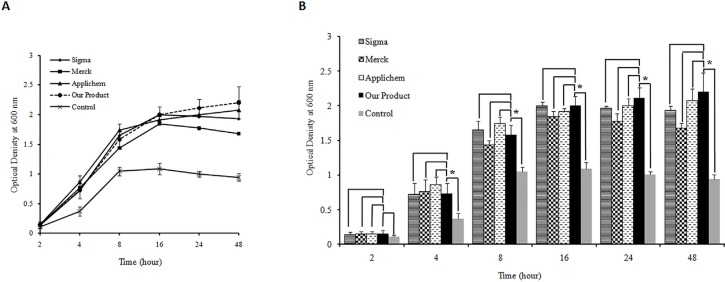
A) Bacterial cell growth profile for *E.* *coli* DH5α as gram negative bacteria on Luria Bertani broth media prepared by the yeast extract produced in this study and commercial products (Applichem, Merck and Sigma). Yeast extract free medium was used as control. Triplicate determinationswere performed for each data point.For the purpose ofclarity the standard deviations are shown only for the medium prepared by our produced yeast extract as well as control media. (B) Histogram of *E.* *coli* DH5α growth in different LB medium. The results indicate that there is no significant difference between growth rate of bacteria in LB broth medium supplemented by the produced yeast extract and LB broth media containing three commercialyeast extracts (p-value >0.05). The analysis shows a significant difference between the growth rate of *E.* *coli* DH5αon the LB medium prepared by our produced yeast extract and control after 4, 8, 16, 24 and 48 h of inoculation (p-value <0.05). The significant differences are marked by asterisks (*).

The quality of the produced yeast extract was also assessed as one of the LB broth media components and was compared with three commercial yeast extracts. The results showed that both type of bacteria were capable of growing in LB media prepared by the produced yeast extract comparable to that in LB media prepared using three commercial yeast extracts (Figures 3a. and 4a.). The statistical analyses indicated that there are no significant differences between growth rates of both type of bacteria in LB broth medium prepared either by our yeast extract product and three commercial ones. Comparing the bacterial growth rate in LB media prepared using the produced yeast extract with growth rate in control media devoid of any yeast extract revealed significant difference for both type of bacteria. The difference was observed at 4–48 h after inoculation of *E. coli *and 8–48 h for *Staphylococcus aureus *(p-value <0.05) (Figures 3b. and 4b.).

*Analyses of yeast extract composition*

Quantitative analyses of the produced yeast extract powder showed that the extract contains: protein (30%), fat (0.42%), sodium chloride (0.67%), ash (12.18%), total volatile nitrogen (9.2%) with moisture of 4.72% and pH of 6.29.

## Discussion

Yeast extract is the product of yeast cells, containing amino acids, lipid, vitamins, minerals and other soluble components withplenty of applications in food industry as well as nutritional source for microbial culture in molecular biology research area and fermentation industry (20-22).The two common approaches for yeast extract production are autolysis and hydrolysis, however, this is not a comprehensive categorization and many methods fall outside of this classification. Autolysis, a natural event occurring after cell death, is self-degradation of the cellular constituents of a yeast cell by the released endogenous digestive enzymes. Several strategies have been reported to improve the autolysis process such as changing the temperature, pH, and osmotic pressure, X-rays irradiation and mechanical comminuting or employing chitosan, chemical inducers, like organic solvents and common salts (23-26). Autolysis process is associated with some disadvantages such as low extraction yield, difficulty in solid-liquid separationdue to risk of microbial contamination resulted from long term incubation. In hydrolysis method, different exogenous enzymes, acids or alkalis are used to disrupt the cell contents (27-29). By applying the exogenous enzymes in hydrolysis process the cost of manufacturing will be increased, moreover, using acids or alkalis in this process will complicate the purification processes.

**Figure 4:**
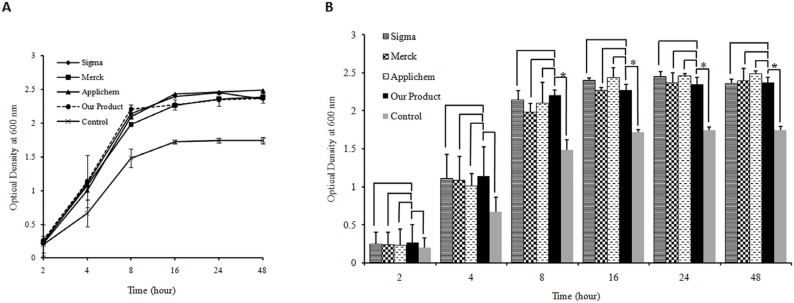
(A) Bacterial cell growth profile for *Staphylococcus* *aureus* as gram positive bacteria on Luria Bertani broth media prepared by the yeast extract produced in this study and commercial products (Applichem, Merck and Sigma). Yeast extract free medium was used as control. Triplicate determinationswere performed for each data point.For the purpose ofclarity the standard deviations are shown only for the medium prepared by our produced yeast extract as well as control media. (B) Histogram of *Staphylococcus* *aureus* growth in different LB medium. The results indicate that there is no significant difference between growth rate of bacteria in LB broth medium supplemented by the produced yeast extract and LB broth media containing three commercialyeast extracts (p-value >0.05). The analysis shows a significant difference between the growth rate of *Staphylococcus* *aureus* on the LB medium prepared by our produced yeast extract and control after 8, 16, 24 and 48 h of inoculation (p-value <0.05). The significant differences are marked by asterisks (*).

Studies show that various mechanical procedures have been employed in some protocols to improve the auto-or hydrolysis processes, but they have not been used alone for yeast extract production (30-31). In the current work, we developed a fast mechanical method to produce the yeast extract from baker’s yeast (*Saccharomyces cerevisiae*) in order to be used as nutrient agent in bacterial culture media. In this method, high temperature and pressure were applied to the suspension of yeast cells followed by rapid cooling. It triggers loss of cell membrane integrity by inducing a shock for yeast cells, which cannot be regarded as either autolysis or hydrolysis based method. Subsequently, the soluble fraction of the extract was subjected to spray dryer for obtaining yeast extract in the powder form, which is more suitable for storage. The quality of the produced yeast extract was very promising concluded from the growth pattern of bacterial cells in media prepared from this product. It is important to note thatthe produced yeast extract was capable of being used by bacteria as a sole source of energy in bacterial solid and liquid media (Figures 1. and 2.). The produced yeast extract is comparable with the three commercial yeast extracts in terms of bacterial growth properties in LB media (Figures 3 and 4). The advantages of our developed method for the production of yeast extract compared to the existing protocols are as followings: 

*i) *No chemicals like acids, alkalis or salts were used in extraction process, which in turn eliminates complication in the downstream processes due to removing the added reagents, and decreases the cost of production.

*ii) *In extraction process, no enzyme was used and therefore the final cost of production would be reduced.

*iii) *The number of required steps in the extraction process was decreased which speeds up the yeast extraction production compared to the other available tedious and time consuming methods. 

## Conclusion

In summary, we developed a simple, fast and cost benefit method for producing yeast extract from baker’s yeast (*Saccharomyces cerevisiae)*. This method can be used in research laboratories to reduce the research costs, and scaled up to produce large quantities for industrial applications where microbial culturing is needed.

## Conflict of interest

The authors have no conflict of interest to declare.
